# Can a framed intervention motivate older adults in assisted living facilities to exercise?

**DOI:** 10.1186/s12877-019-1060-z

**Published:** 2019-02-18

**Authors:** Jari Vanroy, Jan Seghers, Jannique van Uffelen, Filip Boen

**Affiliations:** KU Leuven, Department of Movement Sciences, Tervuursevest 101, 3001 Leuven, Belgium

**Keywords:** Aging, Physical activity, Message framing, Self-determination theory

## Abstract

**Background:**

The majority of institutionalized older adults do not exercise, despite the many health benefits. The current study investigated whether a framed intervention can motivate older adults in assisted living facilities (ALFs) to perform functional resistance exercises. It was hypothesized that repeated framing of these exercises from a prevention perspective (e.g., to avoid health deterioration) would nurture the development of controlled motivation to exercise. By contrast, repeated framing of the exercises from a promotion perspective (e.g., to improve health) was expected to lead to higher exercise frequencies over time and to foster the development of autonomous motivation. Autonomous motivation was hypothesized to predict higher exercise frequencies over time.

**Methods:**

A total of 111 residents, aged 65+ years (*M* = 81.4 y; *SD* = 6.4 y) participated in the study. These participants received a printed three-week individual program with a standard session of eight functional resistance exercises. Four weekly sessions were recommended. Participants were semi-randomized into three framing conditions: neutral (i.e., control), prevention or promotion. They received condition-specific written and spoken messages about the exercises at the beginning of the intervention. The spoken messages were repeated at the end of each week. Participants kept a checklist with their weekly exercise frequency and at corresponding points in time, they completed a questionnaire about their levels of autonomous and controlled motivation to exercise.

**Results:**

Across conditions and time points, the exercise frequencies and the levels of autonomous motivation were generally high, whereas the levels of controlled motivation were generally low. Contrary to the expectations, there were no significant framing effects. However, higher levels of autonomous motivation predicted higher exercise frequencies. During the final exercise week, this was especially the case for intrinsic regulation (i.e., for the sake of the activity).

**Conclusions:**

This study indicates that older adults who live in ALFs can be motivated to perform functional resistance exercises. Given the importance of intrinsic regulation, we advise to create an exercise atmosphere that allows for immediate, positive experiences and in which the basic psychological needs for autonomy, competence and relatedness are satisfied.

**Trial registration:**

ClinicalTrialsID NCT02780037 (23 February 2016).

## Background

The world population is ageing because of decreased fertility rates and increased life expectancy [1]. It is estimated that in 2050, more than 4 % of the whole population will be 80 years or older, compared to only 1 % in 2002 [2]. In older people, physical activity is associated with lower mortality rates [3] and enhanced physical and mental health [4].

Physical activity refers to any bodily movement produced by skeletal muscles and incorporates exercise [5]. Exercise includes a planned, repetitive component as well as a fitness goal [5]. Typical examples of exercise are aerobic activities to enhance cardiorespiratory fitness and resistance exercises to enhance muscular strength. In order to achieve the health benefits from physical activity, the World Health Organization recommends that people aged 65+ years perform a combination of vigorous and moderate aerobic activities of at least 75–150 min, as well as muscle strengthening activities on two or more days a week [4]. Although some studies suggest benefits of lighter aerobic physical activities [6, 7], recommendations for resistance exercises remain fairly uncontested [8].

Despite these recommendations, exercise participation among older people is low, particularly with respect to resistance exercises and for people who live in an institution [9, 10]. Even in institutionalized older adults, exercise (in this case ergometer cycling) has been shown to benefit functional performance and muscular strength, given a sufficient participation [11]. Hence, it is important to study whether and how institutionalized older adults can be motivated to perform (resistance) exercise.

Older people face several barriers to exercise, including health issues [3, 12, 13] and a lack of knowledge about the relationship between exercise and health [3, 13]. On the other hand, older people do expect certain benefits from exercise, such as social interaction [12, 14], competence mastery [14], physical health and enjoyment [12]. A systematic review showed that exercise adherence rates are higher in older people who live alone, who have higher socioeconomic status, better physical health, better physical and cognitive abilities and fewer depressive symptoms [15]. Despite this general understanding of determinants to exercise in older people, theory-based motivational research in this population is lacking. Nevertheless, several motivational theories are available, which have been applied successfully in the exercise context but primarily among younger populations.

A first line of studies is based on the Self-Determination Theory (SDT) [16] and its distinction between autonomous and controlled forms of motivation. *Autonomous* forms of motivation are characterized by a high degree of self-determination and include the following three behavior regulation styles, ranking from more to less self-determined: intrinsic regulation, (i.e., driven by positive activity experiences, such as enjoyment during exercise); integrated regulation (i.e., driven by identity evaluations, such as a fit lifestyle); and identified regulation (i.e., driven by personal values, such as health). These autonomous regulation styles are assumed to result in sustained behavior.

By contrast, *controlled* forms of motivation are characterized by a low degree of self-determination and include the following two behavior regulation styles, ranking from more to less self-determined: introjected regulation (i.e., driven by internal pressures, such as guilt); and external regulation (i.e., driven by external pressures, such as a sanction).

SDT has been adopted as a guiding framework to explain a diverse range of behaviors, including exercise. However, the majority of studies were conducted in younger samples, such as college students [17, 18]. Of the studies that specifically targeted older adults, the samples were relatively younger and fitter [19–21]. Nonetheless, the general applicability of the SDT-framework among older people has been illustrated by Vallerand and O’Connor through a narrative review [17]. These authors concluded that the most prominent forms of motivation in older people across several important life domains were autonomous ones, especially among women. However, there was a difference between younger and older people; in older people non-intrinsic autonomous forms of motivation (no further division possible) were more strongly correlated with certain positive outcomes (e.g., less depression) than intrinsic motivation.

A second line of studies is based on the distinction between prevention versus promotion motives [22–24]. Prevention motives are regulated by a desire to maintain the status quo (e.g., to stay healthy). By contrast, promotion motives are regulated by a desire to improve the status quo (e.g., to get healthier). In general, promotion motives have been found to lead to sustained behavior, including exercise ([25–27], p. 66). Some studies (e.g., [23, 28]) have argued that the motivational power of promotion motives only holds when a number of conditions are met. One of these conditions is a chronic promotion orientation, which refers to a general inclination towards promotion motives [23]. Another condition is a low-risk task [28], which refers to a behavioral choice (e.g., to exercise or not) that is unlikely to yield a certain envisioned negative outcome (e.g., heart failure) at a given point in time. However, the current study does not focus on these conditions but on the distinction between prevention and promotion motives in general.

It is important to test the predictive value of the distinction between prevention and promotion motives in older people, especially those who live in an institution. Their day-to-day communication is overwhelmed with prevention language, such as having to take medications to prevent health risks (e.g., heart failure). To illustrate this, Lockwood and colleagues tested the effects of positive and negative role models on the strength of health-related motivation in younger (i.e. 18–25 years) and older (i.e., 60–75 years) adults [29]. Positive role models are people who do well at a certain envisioned dimension (e.g., healthy people) whereas negative role models do not (e.g., ill people). Both younger and older adults were motivated by positive role models. However, older adults were also motivated by negative role models, whereas younger adults were not. These differences were partially mediated by differences in health-related prevention orientation. Interestingly, although promotion orientations were higher in the younger age group than in the older age group, both younger as well as older adults were more promotion than prevention oriented.

Both distinctions (i.e., autonomous/controlled and prevention/promotion) might share functional overlap. For example, imagine a woman aged 80 years who takes her medications because her doctor told her that she has to in order to prevent heart failure. In that case, it is unclear whether controlled and prevention motives can and should be disentangled. To illustrate, the wording ‘have to’ has two typical interpretations. The first interpretation is a social one, which gives rise to controlled motivation. For example, a child ‘has to listen to his parents’. The second one is a preventive one, which gives rise to prevention motivation. For example, a child ‘has to brush his teeth’. As a consequence, it is not clear either whether in the example above the woman’s behavior and affect are consequences of the controlled aspect of her motivation or of the prevention aspect. A first step towards clarification is to exame to what extent prevention/promotion and autonomous/controlled motives predict each other as well as a targeted behavior.

Promising preliminary attempts in this direction have already been made. For example, a cross-sectional survey showed that self-determined motivation (i.e., autonomous relative to controlled motivation) to exercise was predicted positively by mastery-approach goals [30]. These mastery-approach goals align with promotion motives (e.g., to learn or improve as much as possible). However, such attempts are still in their infancy and have not yet been applied to the population of older adults.

The current study has two general aims. The first aim is to examine whether older adults can be motivated to perform functional resistance exercises. To this end, the feasibility of a newly developed framed intervention will be evaluated in an exploratory way, through measures of attendance and exercise frequency.

The second aim is to examine the mechanism underlying an older adult’s motivation to exercise. It is presumed that both promotion framing (as a means to induce promotion motivation) as well as autonomous motivation lead to higher exercise frequencies over time. In addition, prevention framing (as a means to induce prevention motivation) is supposed to lead to controlled motivation to exercise, whereas promotion framing is supposed to lead to autonomous motivation to exercise. Hence, the following three confirmatory hypotheses will be tested. First, we predict that older adults who receive repeated promotion framing will exercise more during the final exercise week than older adults who receive repeated prevention framing or no specific framing (H1). Second, we predict that older adults who receive repeated prevention framing will develop higher levels of controlled motivation to exercise than older adults who receive repeated promotion framing or no specific framing (H2a); by contrast, older adults who receive repeated promotion framing will develop higher levels of autonomous motivation to exercise than older adults who receive repeated prevention framing or no specific framing (H2b). Third and finally, we predict that higher levels of autonomous motivation to exercise predict higher exercise frequencies among older adults (H3).

To test these hypotheses, older adults who lived in an assisted living facility (ALF) were offered a three-week exercise intervention. Several times over the three-week time span, prevention and promotion motives were framed while exercise frequencies and levels of autonomous and controlled motivation were measured. This approach enabled us to differentiate between initial effects (i.e., after 1 week with a single framing) and effects over time (i.e., after 3 weeks with repeated framings). This differentiation has two advantages. First, it is important to know in practice how to motivate older adults not only to start exercise participation but also to continue exercise participation. Second, the differentiation is theoretically grounded in research that distinguishes between the usefulness of initial motives and evolved motives [31, 32]. Moreover, a walking intervention study in older people showed that over a four-week time span, positively framed messages (e.g., ‘walking is associated with better subjective health’) promoted walking more effectively than negatively framed messages (e.g., ‘not walking is associated with worse subjective health’) [33].

## Methods

### Design

The current intervention took place in eight ALFs in Flanders between February and June 2016. There were three framing conditions: a neutral condition (N), a prevention condition (PV) and a promotion condition (PM). These conditions were present in each ALF. Within each ALF, participants were allocated to three groups of equal size (i.e., conditions) of two to nine persons, who were treated together for practical reasons and to prevent spillover. This assignment was not completely at random considering that we took into account participants’ schedule limitations (e.g., not available in the afternoon) and partnerships (i.e., partners were put in the same condition). Moreover, we had to ensure similar numbers of participants per condition across ALFs (i.e., in the total sample). This approach implies that a semi-randomized controlled trial was adopted.

Researchers (i.e., test leaders) and participants met four times (t1, t2, t3 and t4) with weekly intervals (6–8 days). These four meetings included the measurement moments, three of which were formally used in the current study (i.e., t1, t2 and t4).

All participants received a printed three-week functional resistance exercise program with the necessary information to perform it on their own (see further). Participants in the PV and PM additionally received a condition-specific framing of the outcomes they could expect when they performed the exercise program (see further). In some cases, participants in the N asked for a rationale behind their participation. In those cases, the scientific value of participation in the project was stressed. If this answer was not satisfactory, some functional associations with exercise were mentioned in a brief and neutral way (e.g., exercise is related to health).

The four meetings were held at participants’ ALF and for each condition separately. These meetings took place in a group format, apart from a few exceptions (e.g., for a participant who was ill we organized an individual session). Each meeting lasted for about 1 hour and included the condition-specific framing (PV and PM only) and a questionnaire (see further). During the first meeting, the exercise program, the weights and the exercise frequency checklist were administered and explained (see further). During the last meeting, the weights and checklist were collected. The same order of meetings was used within and across ALFs: first the N, then the PV and finally the PM. Table 1 summarizes the study design.

**Table 1:** Study design

	N	PV	PM
t1 meeting	• Measurements • Explanation checklist and program	• Measurements • Explanation checklist and program • Framing	• Measurements • Explanation checklist and program • Framing
*Week 1 exercise program (6–8 days)*			
t2 meeting	• Measurements	• Measurements • Framing	• Measurements • Framing
*Week 2 exercise program (6–8 days)*			
t3 meeting	• Measurements^a^	• Measurements^a^ • Framing	• Measurements^a^ • Framing
*Week 3 exercise program (6–8 days)*			
t4 meeting	• Measurements	• Measurements • Framing	• Measurements • Framing

^a^These measurements were not used in the analyses

### Participants

Participants were recruited in two steps. In the first step, 16 ALFs were informed about the general aims and the practical details of the study and invited to participate. Eight of these ALFs decided to participate in the study and eight did not because of a lack of potential participants (3x), a lack of time (i.e., in the startup phase; 2x), an inappropriate population (i.e., dementia; 1x) or for unknown reasons (i.e., no response; 2x). In the second step, residents (or visitors) of the participating ALFs were invited to participate. The way in which people were invited to participate differed between ALFs; for example, in some ALFs people were invited personally by a staff member of the ALF (i.e., a nurse or board member) whereas in others an information session was given by a researcher. Nonetheless, we tried to ensure that all eligible people were aware of the study and that external motivation was kept minimal (i.e., standardized information). People were eligible if they were aged 65 years or older and free from severe mental (e.g., dementia) and physical (e.g., wheelchair-bound) impairment, as determined by a staff member of the ALF. A total of 111 people enrolled in the study. The number of participants per ALF varied between 7 and 25. After enrollment, participants in each ALF were semi-randomly assigned to one of the three conditions (N, PV or PM). All participants signed an informed consent form. This study was approved by the Medical Ethics Committee UZ KU Leuven (S58680).

### Intervention

#### Exercise program

The exercise program lasted for 3 weeks and consisted of a standard session of approximately 20 minutes that included eight [8] functional resistance exercises (e.g., standing from a chair). We advised to perform at least four sessions per week, with a minimal interval of 12 hours between two sessions. We also advised to increase the number of repetitions per exercise each week (e.g., week 1: 8, week 2: 10, week 3: 2 × 8). The exercises targeted different body areas, were demonstrated by a test leader (i.e., BSc/MSc in kinesiology/physiotherapy) and allowed for differentiation (i.e., individualized variations), for example with respect to the range of motion. The exercises could be performed alone or in a group, with or without two external ankle/wrist weights. These weights (half a kg each) were made available to all participants for three weeks at no cost. Participants could ask questions about the exercise program and the execution of the exercises at every meeting. This approach had the additional advantage that exercise participation could be conceptualized as a sequence of categorical choices.

#### Prevention framing

In the PV, the benefits of the program were expressed (i.e., framed) by the test leaders as outcomes (losses) that could be prevented. These outcomes consisted of a general statement of a worse situation, a loss in muscle mass and muscle function and five specific outcomes as a result of these decreases, each accompanied by an example. These five outcomes were: (1) ‘independence’, because of the significance for older adults, and two typical internal and external goals. The internal goals were (2) ‘health’ and (3) ‘social capabilities’; the external goals were (4) ‘appearance’ and (5) ‘status’. We decided to include both internal and external goals to account for the differential impact of these kinds of goals [16].

Different Dutch terms were used to convey the messages of loss (e.g., decrease, worsening …) and prevention (e.g., avoid, counter …). In addition, different communication channels were used, namely: (1) (shortened) spoken descriptions, (2) visual verbal information on the checklist, (3) visual verbal and symbolic (i.e., an arrow pointing down) information on a reminding piece of paper, and (4) a specific program title, translated as ‘Fully Against Force Decline!’ To ensure the repeated framing, the spoken descriptions were stated by the test leaders at every meeting. In addition, the checklist also functioned as a measurement tool to keep record of (see further).

#### Promotion framing

In the PM, the benefits of the program were expressed (i.e., framed) by the test leaders as outcomes (gains) that could be attained (as opposed to prevented). These outcomes consisted of a general statement of a better situation, a gain in muscle mass and muscle function and the same five specific outcomes as in the PV, framed in promotion terms (e.g., health improvement instead of the prevention of health deterioration). Different Dutch terms were used to describe the concept of gain (e.g., progress, amelioration …) and different communication channels were used, in analogy with the prevention condition. The specific program title can be translated as ‘Full Force Ahead!’ To ensure the repeated framing, the spoken descriptions were stated by the test leaders at every meeting. In addition, the checklist also functioned as a measurement tool to keep record of (see further).

### Measurements

The reasons for absence at the meetings and for drop-out were based on self-report by participants and/or staff members of the ALFs. Exercise frequency was determined by means of a checklist (see further), handed at the first meeting and to be kept by the participants themselves. Baseline characteristics and levels of autonomous and controlled motivation (see further) were determined by means of a paper-and-pencil questionnaire. The questionnaire was handed and explained at each meeting by the test leaders and had to be filled out by each participant during the meetings (or in some rare cases, at home). Due to illness, one participant completed her last questionnaire a few days after the final meeting. The questionnaire comprised a few additional psychological questions but most participants were able to fill out the entire questionnaire within approximately 20 minutes.

### Exercise frequency

Participants received a checklist to keep record of their exercise sessions. This checklist was explained at the first meeting. The checklist was divided into three rows, one for each week, and seven columns, one for each day of the week (20–22 days in total). Participants had to check a cell after completion of an exercise session. Because several participants encountered a specific limitation that made it hard to perform a specific exercise (e.g., right shoulder impairment), an exercise session could also be checked if a participant performed only six or seven out of the eight exercises. In addition, participants were asked to make mention of the days they were ill/injured and the days they performed alternative physical activities of at least 15 minutes. At the meetings we asked participants whether the checklist was feasible to use and assisted them if necessary.

#### Baseline characteristics

Participants had to fill out their date of birth and their sex. The questionnaire also included one item to assess participants’ subjective level of physical health (i.e., “I feel physically healthy”) and one to assess their subjective level of mental health (i.e., “I feel mentally healthy”). Participants could answer on a seven-point Likert scale ranging from 1 (‘completely not agree’) to 7 (‘completely agree’).

#### Autonomous motivation

The questionnaire contained nine items to assess participants’ levels of autonomous motivation to performing the exercises. These items were based on the third version of the Behavioral Regulation in Exercise Questionnaire (BREQ-3) [34, 35] and comprised three items for each of the subscales (i.e. intrinsic, integrated and identified regulation). Participants could answer on a seven-point Likert scale ranging from 1 (‘completely not agree’) to 7 (‘completely agree’). All items were initiated by the question: “Why do you perform the exercises from the program?” An example item was: “Because I enjoy it” (intrinsic regulation). Internal consistencies were measured across conditions and were deemed acceptable at all measurement points (t1, t2 and t4): autonomous motivation, *α*s = .90–.94 (*n* = 75–81); intrinsic regulation, *α*s = .91–.95 (*n* = 76–90); integrated regulation, *α*s = .81–.90 (*n* = 76–85); identified regulation, *α*s = .76–.77 (*n* = 78–94).

#### Controlled motivation

The questionnaire contained seven items to assess participants’ levels of controlled motivation to performing the exercises. Three items measured external regulation and were based on the BREQ-3. The four other items measured avoidance oriented introjected regulation (two items) and approach oriented introjected regulation (two items) and were based on the Multidimensional Work Motivation Scale [36]. Participants could answer on a seven-point Likert scale ranging from 1 (‘completely not agree’) to 7 (‘completely agree’). All items were initiated by the question: “Why do you perform the exercises from the program?” An example item was: “Because I want to be proud of myself” (approach oriented introjected regulation). Internal consistencies were measured across conditions and deemed acceptable at all three measurement points: controlled motivation, *α*s = .87–.89 (*n* = 78–91); external regulation, *α*s = .86–.87 (*n* = 78–96); introjected regulation, *α*s = .77–.82 (*n* = 78–91); avoidance oriented introjected regulation, *α*s = .86–.92 (*n* = 78–93), approach oriented introjected regulation, *α*s = .69–.85 (*n* = 79–95).

### Analysis

SPSS 24.0 was used for statistical analyses. Because data were skewed for many variables, even after some common transformations, non-parametric tests were performed. Associations between variables were assessed using Spearman rank correlations. Differences between conditions in raw scores and in change scores were assessed using Mann-Whitney U tests. Differences between conditions in proportions were assessed using Chi^2^ tests. Significance levels were set two-sided at *p* < .05. No corrections for multiple testing were used. Missing values were deleted pairwise.

## Results

### Sample

Participants were on average 81.4 years old (*N* = 99; *SD* = 6.4 years) and about two-thirds were female (67.3%). Participants generally felt in good physical (*N* = 97; *M* = 5.4; *SD* = 1.4) and mental health (*N* = 97; *M* = 5.9; *SD* = 1.2). The baseline characteristics per condition are presented in Table 2. There were no significant differences between the conditions.

**Table 2:** *Baseline characteristics*

	N	PV	PM
*n* ^a^	38	37^b^	36
Age (years; M ± SD)	80.6 ± 7.3 (*n* = 36)	81.4 ± 6.9 (*n* = 32)	82.4 ± 4.6^c^ (*n* = 31)
Sex	24♀ 14♂ (*n* = 38)	26♀ 10♂ (*n* = 36)	24♀ 12♂ (*n* = 36)
Physical health (1–7; M ± SD)	5.4 ± 1.4 (*n* = 36)	5.5 ± 1.5 (*n* = 29)	5.3 ± 1.4 (*n* = 32)
Mental health (1–7; M ± SD)	5.9 ± 1.2 (*n* = 36)	5.7 ± 1.4 (*n* = 29)	6.0 ± 1.0 (*n* = 32)

^a^Two partners were accidently divided into a different condition (1 N, 1 PM). Given the fact that the one in the N dropped out at t1, this deviation was not considered problematic

^b^Two participants (PV) started one day later than the other participants in their ALF. These two participants received the instructions and materials from a fellow participant in the same ALF and condition, with whom we had discussed the procedure the day before

^c^One participant still had to become 65 during the course of the year

Note: No significant differences were found between conditions (*p*s > .05)

### Attendance

During the course of the study, 72 out of the 111 participants (64.9%) were present at all meetings. However, some participants were not able to be present at one (or two) meeting(s) due to practical (*n* = 8) or medical (*n* = 3) issues. In addition, some participants discontinued participation at t1, t2 or t3. The rates and reasons of drop-out are shown in Table 3. The majority of the 29 participants who dropped out, did so at/before the beginning of the study (i.e., at t1; *n* = 12) or after 1 week (i.e., at t2; *n* = 14). The most frequently cited reason for drop-out was by far a medical issue (twelve times in total).

**Table 3:** Rates and reasons of drop-out

	N	PV	PM
t1	*n* = 2 (5%; 0♀ 2♂) • Practical (2x)	*n* = 7 (19%; 4♀ 2♂) • Practical (4x) • Medical (3x) • Motivation, age^a^, pain (1x each)	*n* = 3 (8%; 3♀ 0♂) • Motivation, age, medical (1x each)
t2	*n* = 0	*n* = 4 (11%; 4♀ 0♂) • Age, medical (2x each) • Pain, practical (1x each)	*n* = 10 (28%; 6♀ 4♂) • Medical (5x) • Motivation, pain (2x each) • Age, other exercise (1x each)
t3	*n* = 1 (3%; 1♀ 0♂) • Other exercise (1x)	*n* = 1 (3%; 1♀ 0♂) • Age, pain (1x each)	*n* = 1 (3%; 1♀ 0♂) • Medical (1x)
total	*n* = 3 (8%; 1♀ 2♂) • Practical (2x) • Other exercise (1x)	*n* = 12 (32%; 9♀ 2♂) • Medical, practical (5x each) • Age (4x) • Pain (3x) • Motivation (1x)	*n* = 14 (39%; 10♀ 4♂) • Medical (7x) • Motivation (3x) • Age, pain (2x each) • Other exercise (1x)

^a^Some participants considered themselves too old to participate

Notes: Several reasons per participant were allowed

One participant’s (PM) reason of drop-out was unknown

The drop-out rates were significantly lower in the neutral condition than in the other two conditions at t2 as well as in total (*p* < .05). The N also showed marginally significantly lower drop-out rates than the PV at t1 (*p* = .09). There were no significant differences in sex or controlled motivation at t1 between participants who dropped out (at t2 or in total) and participants who did not. However, participants who dropped out (in total) were marginally significantly (*p* = .10) older than participants who did not (*N* = 99; median of 84 years compared with 82 years). In addition, participants who dropped out at t2 showed marginally significantly (*p* = .07) lower levels of autonomous motivation at t1 than participants who did not (*N* = 96; median of 5.6 compared with 6.1 on a 1–7 scale).

### Exercise frequency

Table 4 depicts the median number of weekly exercise sessions in each condition at t2, with respect to the first exercise week, and at t4, with respect to the third exercise week. In general, these median numbers reached the recommended amount of four exercise sessions per week (4.0–5.0).

**Table 4:** Median exercise frequencies

Exercise sessions	NC	PV	PM
t2	5.0 (4.0–7.0)	5.0 (4.0–7.0)	4.0 (3.0–6.0)
t4	5.0 (4.0–7.0)	4.0 (2.5–6.0)	5.0 (2.5–7.0)

Notes: N: *n* = 34–35; PV: *n* = 25–27; PM: *n* = 24–25

No significant differences were found between conditions (*p*s > .05)

The 25–75 percentile ranges are given between brackets

In the first exercise week (i.e., at t2), 68 out of 85 participants (80.0%) performed at least four exercise sessions, 25 participants (29.4%) performed the maximum number of seven exercise sessions and six participants (7.1%) did not perform an exercise session. In the final exercise week (i.e., at t4), 58 out of 80 participants (72.5%) performed at least four exercise sessions, 22 participants (27.8%) performed seven exercise sessions and seven participants (8.8%) did not perform an exercise session.

### Framing effects on exercise frequency

There were no significant differences in weekly exercise frequency between any two conditions at either point in time. However, the exercise frequency at t2 was marginally significantly lower in the promotion condition than in both other conditions (N: *p* = .07; PV: *p* = .1).

### Framing effects on controlled/autonomous motivation

Table 5 displays the median levels of autonomous and controlled motivation for each condition at t1, t2 and t4. In general, the median levels of autonomous motivation were rather high (5.6–6.2) whereas the median levels of controlled motivation were rather low (2.4–3.2). There were no significant differences in either autonomous or controlled motivation between any two conditions at any point in time. However, the levels of controlled motivation at t4 were marginally significantly (*p* = .08) higher in the neutral condition than in the promotion condition.

**Table 5:** Median levels of autonomous and controlled motivation

	N		PV		PM	
	AM	CM	AM	CM	AM	CM
t1	6.2 (5.5–6.7)	3.2 (2.4–4.4)	6.2 (5.4–6.7)	2.7 (2.1–3.8)	5.7 (5.0–6.2)	3.0 (2.1–4.1)
t2	6.0 (4.9–6.5)	2.8 (2.3–3.8)	6.1 (4.9–6.6)	2.7 (1.9–4.0)	5.6 (4.7–6.1)	2.4 (1.4–4.2)
t4	5.9 (4.7–6.4)	3.1 (2.4–5.0)	5.8 (4.8–6.4)	3.0 (2.0–4.6)	5.6 (4.6–6.3)	2.7 (1.9–4.1)

Notes: *AM* autonomous motivation (1–7), *CM* controlled motivation (1–7)

higher scores indicate higher levels of autonomous/controlled motivation

No significant differences were found between conditions (*p*s > .05)

N: *n* = 33–36; PV: *n* = 23–30; PM: *n* = 21–32

The 25–75 percentile ranges are given between brackets

As a next step, within-participant changes in levels of autonomous and controlled motivation were calculated at short (t2 – t1) and longer (t4 – t1) term. These changes were then compared between the conditions. Again, there were no significant differences in changes in either autonomous or controlled motivation between any two conditions at either time interval. Nevertheless, participants in the PM did show smaller declines in autonomous motivation and smaller increases in controlled motivation than participants in the PV. However, in the PV, autonomous motivation levels at t1 were relatively high and controlled motivation levels at t1 were relatively low, whereas the opposite pattern was found in the PM (i.e., relatively low levels of autonomous motivation and high levels of controlled motivation at t1).

### Effects of controlled/autonomous motivation on exercise frequency

Table 6 shows the associations between controlled/autonomous motivation levels and exercise frequencies across conditions. Higher exercise frequencies at t2 were predicted by higher levels of autonomous motivation both at t1 (*ρ* = .28; *p* = .01) and at t2 (*ρ* = .28; *p* = .02). Higher exercise frequencies at t4 were predicted by higher levels of autonomous motivation at t4 (*ρ* = .22; *p* = .05).

**Table 6:** Spearman rank correlations between motivation and exercise frequency

	Exercise sessions t2	Exercise sessions t4
AM t1	.28**	.11
AM t2	.28*	.06
AM t4	.21	.22*
CM t1	−.03	−.03
CM t2	.13	.04
CM t4	.15	.08

*p < .05; **p < .001

Notes: *AM* autonomous motivation, *CM* controlled motivation; *n* = 75–84

Given these significant associations, the subscales of autonomous motivation were compared with the exercise frequencies across conditions. Higher exercise frequencies at t2 were predicted by higher levels of intrinsic regulation at t1 (*ρ* = .28; *p* = .01), at t2 (*ρ* = .23; *p* = .04) and at t4 (*ρ* = .24; *p* = .03), as well as by higher levels of integrated (*ρ* = .29; *p* = .01) and identified (*ρ* = .29; *p* = .01) regulation at t2. Higher exercise frequencies at t4 were only predicted by higher levels of intrinsic regulation at t4 (*ρ* = .26; *p* = .02).

## Discussion

This study investigated whether and how older adults in ALFs could be motivated to exercise through a framed intervention that included functional resistance exercises. The effects of repeated exercise framing on exercise frequency and controlled/autonomous were examined, as well as the relation between exercise frequency and controlled/autonomous motivation. It was presumed that that both promotion framing and autonomous motivation would lead to higher exercise participation over time, that prevention framing would lead to controlled motivation to exercise, and that promotion framing would lead to autonomous motivation to exercise.

### Aim 1: Can older adults in ALFs be motivated to exercise?

The results indicated that the intervention indeed functioned as a vehicle to motivate older adults in ALFs to exercise. Firstly, a relevant sample of 111 older adults was reached. Their mean age (81.4 years) was high in comparison with other exercise interventions in older adults [37]. Although the majority of participants (67.3%) were female, such a sex imbalance is common among similar studies [17, 20, 29, 37]. Moreover, 74% of ALF residents in the US are female [38].

Secondly, most participants (69.4%) were present at all meetings. Furthermore, during the final two exercise weeks, only three participants dropped out. However, twenty-six participants (23.4%) dropped out in the initial stages of the study, which is rather high in comparison with other exercise intervention studies among older people [39]. The participants who dropped out in the current study were slightly older, less autonomously motivated to exercise and less likely to be in the neutral condition. The most frequently reported reason of drop-out was a medical issue. Remarkably, a practical issue (e.g., on a trip) was also frequently mentioned as a reason of drop-out (seven times in total), but only in the neutral and prevention conditions, not a single time in the promotion condition. The timing of the sessions, which was in a fixed order (i.e., N – PV – PM), might have played a role. It has been shown that older adults, as opposed to younger adults, perform better at memory tasks before noon than after noon [40]. Hence, we advise future researchers to take into account the time of the day when the session takes places. We presume that the time period right after breakfast (e.g., between 10 and 11 a.m.) is generally suitable in institutionalized older adults. At that time, these people are still energized and there is less conflict with other activities (e.g., lunch, nap, card game …).

Thirdly, exercise frequencies and levels of autonomous relative to controlled motivation were generally high. Such high levels of autonomous motivation can be expected among people who voluntarily participate in an intervention study without financial compensation. In addition, high levels of autonomous motivation among older adults are not rare [17]. However, the exercise frequencies were remarkably high (± four average weekly exercise sessions), for example in comparison with the ± two average weekly exercise sessions found in the ergometer cycling intervention among institutionalized older adults [11]. It should be noted however that exercise frequencies in the current study were assessed through unsupervised self-report. It has been shown that older adults are generally inclined to overestimate their activity levels [41]. In the current study however, an exercise session was defined in advance and the checklist was kept by the participants themselves to avoid recall issues. Additionally, it was not possible to evaluate a proper execution of the exercise program during the time it was being performed. Furthermore, a session of eight functional resistance exercises (at maximum) needs relatively little time, energy and other resources to complete, in comparison with a session of most other exercise programs (e.g., ergometer cycling). However, this fact is promising from a practical point of view. This implies that with minimal external resources (e.g., from a staff member or an exercise leader), a growing group of people with health-related risks can be addressed to perform a relevant amount of health-related exercises.

### Aim 2: How can older adults in ALFs be motivated to exercise?

Hypothesis 1 was not supported. Promotion framing did not seem to foster higher exercise frequencies. Although a tendency towards higher exercise frequencies was noticeable in the PM, this tendency did not significantly outweigh initial differences. This tendency might be explained to some extent by regression to the mean and selective drop-out.

Hypothesis 2 was not supported either. Prevention framing did not seem to foster controlled motivation (H2a) and promotion framing did not seem to foster autonomous motivation (H2b). Although there was a tendency towards more autonomous relative to controlled motivation in the PM, this tendency did hardly outweigh initial differences. This tendency might also be explained to some extent by regression to the mean and selective drop-out.

Hypothesis 3 was supported. Autonomous motivation appeared to foster exercise frequency (H3). In particular during the last week, exercise frequency was only predicted by the intrinsic regulation subscale of autonomous motivation. Moreover, initial intrinsic regulation predicted the exercise frequency during the first exercise week. It should be noted though that no strict causality could be inferred because autonomous motivation was not manipulated directly. The value of intrinsic motivation for repeated behavior has already been demonstrated in earlier studies [18] and seems to hold in older adults too. Taken together, these findings align with the existing evidence concerning the usefulness of SDT, as stated in a systematic review of motivational exercise interventions in physiotherapy [42].

### Limitations and future research

The current study paves the way for future studies to expand on the question how older adults can be motivated to exercise in a sustainable way. However, this study had a number of limitations that future research should take into account. First, a three-week period is too short to fully grasp the long-term effects of an exercise intervention based on motivational theory. Although crucial distinctions in motivational processes can be made based on the absence or presence of activity feedback [31] and framing repetition [26], long-term periods in the domain of exercise research typically cover several months. If some initial resistance to change (i.e., exercise) exists, it is probably still somewhat present after 3 weeks. Moreover, such resistance seems most likely under promotion framing, considering that the day-to-day communication of people in ALFs is overwhelmed with prevention language. They might even deem such language to be unrealistic.

Second, a different operationalization of the key intervention constructs might shed another light on the findings of the current study. Examples of a different operationalization include other exercise activities (e.g., walking), different framed outcomes (e.g., only internal/external goals), and a uniform large-scale recruitment procedure. In that way, it will be possible to compare the current study with its many novel elements with other studies that use the same key constructs in different ways. Moreover, this approach allows a reduction of the noise due to a specific operationalization.

Third, a number of relevant outcome measures were not taken into account. For example, more detailed information about non-compliance, experiences during exercise and indicators of well-being should enhance our understanding of which motivational practices are most adaptive and why they are.

Fourth and finally, a number of prominent mediators between the theoretical constructs were not taken into account. These mediators include the basic psychological needs (i.e., autonomy, competence and relatedness), regarding SDT, and the general and domain-specific orientations, regarding the avoidance-approach distinction.

Despite the theoretical relevance, the current study could not demonstrate clear benefits for exercise framing from a promotion perspective, as was predicted. Future research could therefore approach the question whether and how older adults in ALFs can be motivated to exercise from another theoretical point of view (in addition to SDT), namely the Social Identity Approach (SIA) [43]. Based on SIA, it can be expected that older adults can be motivated to exercise within a group of other older adults who exercise too [19, 43–45]. It is however important to recognize that social influences on older adults’ exercise levels can be positive as well as negative [46].

### Implications

The results of the current study imply that exercise leaders should try to make their functional resistance exercise programs enjoyable, also when they deal with older adults. This does not necessarily mean that they should avoid mentioning the benefits of exercise at all costs. However, an extensive and repeated account of these benefits might be interpreted as controlling, in particular in groups where substantial variation in perspectives between different people exists; for example, prevention vs. promotion oriented older adults, intrinsic vs. extrinsic goal oriented older adults. Furthermore, such an emphasis might cause a shift in attribution from intrinsic to more extrinsic forms of motivation.

The results of the current study however do not enable us to draw conclusions on when or whether exercise leaders should adopt prevention versus promotion language to elicit sustainable exercise participation among older adults. Although this does not mean that this differentiation is of no importance, other organizing principles might be more efficient.

Given the importance of intrinsic regulation, we advise to create an exercise atmosphere that allows for immediate [47], positive experiences [48] and in which the basic psychological needs for autonomy, competence and relatedness are satisfied [16]. For example, participants who want can meet once a week to perform their exercises in a group and to discuss their experiences. An expert exercise leader to rely on seems recommended [15]. However, his/her supervision might not be necessary every time.

## Conclusions

Research has shown that exercise is important for health in the hard-to-reach population of older adults. The current study demonstrated that older adults who live in ALFs can be motivated to perform functional resistance exercises. No clear effects from promotion (or prevention) framing were found. However, intrinsic regulation showed positive associations with exercise frequencies.

## Abbreviations

ALF: Assisted living facility
BREQ-3: Behavioral Regulation in Exercise Questionnaire, third version
H: Hypothesis
N: Neutral condition
PM: Promotion (framing) condition
PV: Prevention (framing) condition
SDT: Self-Determination Theory
SIA: Social Identity Approach
